# Expression of *SULTR2;2*, encoding a low-affinity sulphur transporter, in the Arabidopsis bundle sheath and vein cells is mediated by a positive regulator

**DOI:** 10.1093/jxb/ery263

**Published:** 2018-07-19

**Authors:** Sandra Kirschner, Helen Woodfield, Katharina Prusko, Maria Koczor, Udo Gowik, Julian M Hibberd, Peter Westhoff

**Affiliations:** 1 Institute for Plant Molecular and Developmental Biology, Heinrich-Heine-Universität Düsseldorf, Universitätsstraße, Düsseldorf, Germany; 2 Department of Plant Sciences, University of Cambridge, Downing Street, Cambridge, UK

**Keywords:** *Arabidopsis thaliana*, bundle sheath, C_3_ photosynthesis, C_4_ photosynthesis, gene expression

## Abstract

The bundle sheath provides a conduit linking veins and mesophyll cells. In the C_3_ plant *Arabidopsis thaliana*, it also plays important roles in oxidative stress and sulphur metabolism. However, the mechanisms responsible for the patterns of gene expression that underpin these metabolic specializations are poorly understood. Here, we used the Arabidopsis *SULTR2;2* gene as a model to better understand mechanisms that restrict expression to the bundle sheath. Deletion analysis indicated that the *SULTR2;2* promoter contains a short region necessary for expression in the bundle sheath and veins. This sequence acts as a positive regulator and is tolerant to multiple consecutive deletions indicating considerable redundancy in the *cis*-elements involved. It is highly conserved in *SULTR2;2* genes of the Brassicaceae and is functional in the distantly related C_4_ species *Flaveria bidentis* that belongs to the Asteraceae. We conclude that expression of *SULTR2;2* in the bundle sheath and veins is underpinned by a highly redundant sequence that likely represents an ancient and conserved mechanism found in families as diverse as the Asteraceae and Brassicaceae.

## Teaser

Spatial patterning of gene expression in the bundle sheath of Arabidopsis is mediated by a conserved repetitive region in the *SULTR2;2* promoter.

## Introduction

The evolution of multicellularity is associated with individual cell types being able to undertake specialized roles within a tissue. In leaves, bundle sheath (BS) cells form a wreath-like structure around the vasculature, which appears to be analogous to the endodermis of roots (Esau, 1965). The role of BS cells is best characterized in C_4_ species, which partition photosynthesis between the BS and mesophyll cells. In most C_4_ plants, after HCO_3_^−^ is initially fixed into C_4_ acids by phospho*enol*pyruvate carboxylase in mesophyll cells, these C_4_ acids diffuse to the BS where CO_2_ is released and refixed by ribulose-1,5-bisphosphate carboxylase/oxygenase (RuBisCO). Decarboxylation of C_4_ acids in the BS generates a high concentration of CO_2_ around RuBisCO that suppresses the oxygenase activity of the enzyme and in so doing reduces photorespiration (Hatch, 1987). Thus, in C_4_ species, the BS is specialized to allow efficient fixation of CO_2_ in the Calvin–Benson–Bassham cycle. In some C_4_ plants, the BS is also modified in terms of light capture. For example, in maize and sorghum photosystem II does not fully assemble in the BS (Kubicki *et al.*, 1994), but components of cyclic electron transport are more abundant in the BS compared with mesophyll cells (Takabayashi *et al.*, 2005). In addition to these changes associated with photosynthesis, the C_4_ BS is also modified to preferentially undertake starch synthesis and degradation, as well as the initial steps of sulphur assimilation (Friso *et al.*, 2004).

In C_3_ plants, the role of the BS is less clearly defined. It is thought to help maintain hydraulic integrity of the xylem (Sade *et al.*, 2014), regulate flux of metabolites in and out of the leaf (Leegood, 2008) and act as a starch store (Miyake and Maeda, 1976). The C_3_ BS is less important for photosynthesis than that of C_4_ species. However, although only around 15% of all chloroplasts of the C_3_ leaf are found in BS cells (Kinsman and Pyke, 1998), reducing photosynthesis in these cells compromises growth and seed production (Janacek *et al.*, 2009). Thus, although less photosynthetic than the C_4_ BS, this physiological analysis indicates that the BS of C_3_ plants is also specialized. This notion is consistent with analysis of gene expression in this cell type. For example quantification of transcripts available for translation indicate that the Arabidopsis BS is likely important in sulphur metabolism, glucosinolate biosynthesis, trehalose metabolism and detoxification of reactive oxygen species (Aubry *et al.*, 2014). In summary, in both C_3_ and C_4_ plants mechanisms must operate to restrict the expression of some genes to BS cells.

To date, most studies of the mechanisms responsible for preferential gene expression in the BS have used C_4_ species (Hibberd and Covshoff, 2010). In the C_4_ dicotyledon *Flaveria trinervia* the glycine decarboxylase P-subunit (*GLDPA*) gene contains two promoters, one proximal to the coding region and the other more distal. Activity of the distal promoter is high but not cell-type specific. However, in the presence of the proximal promoter, transcripts derived from the distal promoter are degraded in mesophyll cells by nonsense-mediated RNA decay of incompletely spliced transcripts (Engelmann *et al.*, 2008; Wiludda *et al.*, 2012). Despite the phylogenetic distance between the Brassicaceae and the Asteraceae the *GLDPA* promoter from *F. trinervia* is able to generate BS-specific activity in C_3_ Arabidopsis (Engelmann *et al.*, 2008; Wiludda *et al.*, 2012). In *Amaranthus hybridus* 5′ and 3′ untranslated regions (UTRs) of the *RbcS1* gene act to restrict accumulation of the glucoronidase (GUS) reporter to the BS of C_4_*Flaveria bidentis* and appear to function as enhancers of translation (Patel *et al.*, 2006). Lastly, in C_4_*Gynandropsis gynandra* preferential expression of *NAD-ME1* and -*2* genes in the BS is associated with coding sequence rather than UTRs or promoter elements (Brown *et al.*, 2011). The motifs underpinning this regulation are a pair of duons that play a dual role in coding for amino acids as well as the spatial patterning of gene expression associated with the C_4_ leaf. Although these duons are present in C_3_ Arabidopsis and many other land plants they do not act to generate cell-specific expression in the ancestral C_3_ state (Brown *et al.*, 2011; Reyna-Llorens *et al.*, 2018). In summary, current evidence indicates that gene expression in the BS of C_4_ species is controlled by a variety of mechanisms, some of which involve regulatory codes that are derived from the ancestral C_3_ state.

However, our understanding of how gene expression is restricted to the BS in C_3_ species is poor. A small number of promoters including *SHORT-ROOT* (Dhondt *et al.*, 2010), *SCARECROW* (Wysocka-Diller *et al.*, 2000), and *SULTR2;2* (Takahashi *et al.*, 2000) have been reported to drive BS-specific expression in Arabidopsis, but neither the molecular nature of *cis*-regulatory elements controlling their expression, nor transcription factors that control expression in the BS have been identified. An increased understanding of these processes would not only advance our understanding of mechanisms underpinning cell-specific gene expression in multicellular leaves, but also provide insight into whether C_4_ gene expression is built on pre-existing mechanisms found in C_3_ species.

As a step towards uncovering mechanisms that underpin expression in BS cells, we sought to identify mechanisms in *cis* associated with the *SULTR2;2* gene of C_3_ Arabidopsis, which encodes a low-affinity sulphur transporter (Takahashi *et al.*, 2000). Elements in the promoter sequence that regulate the spatial patterning and the strength of gene expression were identified. Specifically, preferential expression in the BS is mediated by a repetitive region that is highly conserved within orthologous genes from species of the Brassicaceae. This region acts to enhance expression in the BS and veins rather than repressing expression in mesophyll cells. Furthermore, the *SULTR2;2* promoter from Arabidopsis generates expression in BS and vein cells in the C_4_ species *F. bidentis* that belongs to the Asteraceae. The most parsimonious explanation for this finding is that a common transcription factor is shared by these phylogenetically dispersed species, and that it functions in both the veins and BS cells of both C_3_ and C_4_ species.

## Materials and methods

### Cloning of promoter–reporter gene constructs and 5′ rapid amplification of cDNA ends

All DNA fragments created by PCR were confirmed by DNA sequencing. A full-length promoter construct was generated via PCR with Arabidopsis Columbia-0 (Col-0) genomic DNA. Subsequent constructs were generated using this as a template. Restriction sites were added to the respective fragments by PCR and fragments were inserted into pBI121 or a partially modified pBI121. Region 2 with internal deletions was synthesized by GenScript and swapped with the full-length region 2 of 2::5.2::*GUS* to generate the internal deletion constructs. Total RNA from leaves of wild type Arabidopsis Col-0 plants was extracted, DNase I treated and purified with the RNeasy® Plant Mini Kit (Qiagen, Hilden, Germany). cDNA was generated from 1 µg RNA and then 5′ rapid amplification of cDNA ends (RACE)-PCR performed using the Advantage® 2 DNA Polymerase Mix (Clontech Laboratories, Mountain View, CA, USA) or Phusion® HF DNA Polymerase (Thermo Fisher Scientific, Waltham, MA, USA). Two nested 3′ oligonucleotides were used, AtSultr2;2–11 and AtSultr2;2–13, both binding in the cDNA of *AtSULTR2;2*. PCR products were cloned and confirmed via colony PCR. Correct clones were the subjects of plasmid preparation and sequencing.

### Transformation and plant growth

Arabidopsis ecotype Col-0 was transformed using floral dipping (Clough and Bent, 1998; Logemann *et al.*, 2006) using *Agrobacterium tumefaciens* strain AGL1. *Flaveria bidentis* was transformed as described previously (Chitty *et al.*, 1999). Successful transformations of Arabidopsis or *F. bidentis* were tested by PCR. Before transplanting to soil, positive transformants of Arabidopsis were selected on kanamycin. Seeds were sterilized by washing twice for 5 min with 20% (v/v) DanKlorix (Colgate-Palmolive, New York, USA) and 0.02% (v.v) Triton X-100 and four times with sterile water. Stratification of seeds was performed at 4 °C for 48 h prior to spreading them on half-strength Murashige–Skoog medium pH 5.7 containing 10 g l^−1^ (w/v) sucrose (Sigma-Aldrich, St Louis, MO, USA), 0.5 g l^−1^ MES (Biomol, Hamburg, Germany), 2.13 g l^−1^ (w/v) Murashige–Skoog basal salts (Duchefa Biochemie, Haarlem, Netherlands), 0.75% agar (SERVA Electrophoresis, Heidelberg, Germany), 50 µg ml^−1^ kanamycin (Sigma-Aldrich) and 100 mg l^−1^ Cefotaxim (Fresenius Kabi Deutschland, Bad Homburg, Germany). Plants were transferred to 14 h light/10 h dark with temperatures of 23 °C day and 20 °C night and a light intensity of 90 µmol m^−2^ s^−1^.

### Visual and quantitative analysis of reporter genes

To take account of effects caused by transgene insertion into different genomic locations, between nine and 41 independent T_0_ plants were analysed for each construct. Histochemical analysis was performed as described previously (Engelmann *et al.*, 2008). For Arabidopsis 3- to 4-week-old rosette leaves or 10- to 14-day-old seedlings and for *F. bidentis* the sixth leaves of 40–50 cm-tall plants were used. Transverse sections were prepared manually using a razor blade. Stained leaves were imaged using light microscopy. Quantification of GUS activity was performed via a fluorimetric assay (Jefferson *et al.*, 1987) using two to four leaves of 3- to 4-week-old T_0_ Arabidopsis plants or the fifth leaf of 40–50 cm-tall T_0_*F. bidentis* plants. For *F. bidentis*, an entire leaf was wrapped in aluminium foil, frozen in liquid nitrogen, pulverized using a hammer, and approximately 300 mg of powdered tissue transferred to 1.5 ml reaction tubes. Arabidopsis leaves were directly harvested into 1.5 ml reaction tubes and frozen in liquid nitrogen. One volume of extraction buffer was added (100 mM Na_2_HPO_4_ pH 7.5, 1 mM EDTA, 0.1% *N*-laurylsarcosine, 0.1% Triton X-100, 20% methanol) and the material homogenized. The homogenate was then centrifuged at 15000 *g* and 4 °C for 5 min, and the clear supernatant used for measurements of protein content and GUS activity. The incubation buffer used to measure GUS activity was identical to the extraction buffer except that 4-methylumbelliferyl-β-D-glucuronide (MUG) was added to a final concentration of 1 mM. The Mann–Whitney *U*-test was used to determine statistical differences between datasets. Imaging of H2B::YFP was performed on a Zeiss LSM 780 confocal laser-scanning microscope, and yellow fluorescent protein (YFP) fluorescence excited at 514 nm with emission detected between 517 and 569 nm.

## Results

**Fig. 1.**
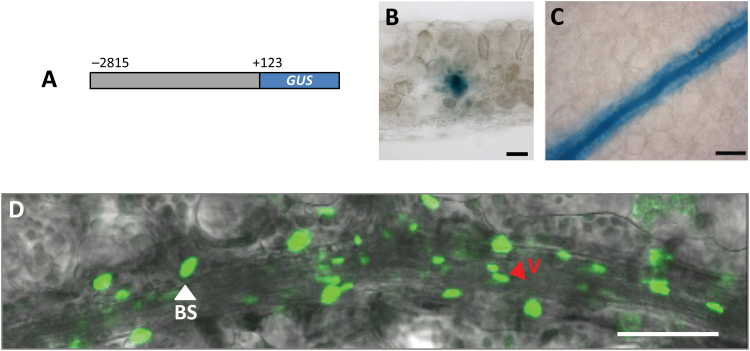
The *SULTR2;2* promoter drives expression in veins and bundle sheath cells of Arabidopsis. (A–C) Nucleotides −2185 to +123 of *SULTR2;2* are sufficient to generate preferential accumulation of the GUS reporter in bundle sheath and vein cells. (D) H2B::YFP fusion marks the larger bundle sheath nuclei (white arrowhead) as well as the smaller, more elongated nuclei of the vasculature (red arrowhead). Histological GUS assays were allowed to proceed for 23 h (B, C). Scale bars: 500 µm (B, C) or 50 µm (D).

Nucleotides −2815 to +123 relative to the predicted *SULTR2;2* translational start site have previously been reported to generate expression in the BS of Arabidopsis (Takahashi *et al.*, 2000). We confirmed this finding (Fig. 1A–C). Staining was evident in vascular tissue as well as the BS but there was no evidence that GUS accumulated in mesophyll cells (Fig. 1A–C). A translational fusion between the YFP and the nuclear localized histone 2B protein under control of the *SULTR2;2* promoter labelled nuclei of BS cells and vascular tissue (Fig. 1D) and indicated that the presence of GUS in the BS and veins was due to gene expression and not diffusion of the dye outwards from to vascular tissue. Consistent with previous reports (Chytilova *et al.*, 1999), it was noticeable that vascular nuclei were elongated and rod-like whilst the BS contained larger, more spherical nuclei (Fig. 1D). We conclude that nucleotides between −2815 and +123 of *SULTR2;2* are sufficient to drive gene expression in vascular and BS cells of Arabidopsis, and thus sought to understand the sequences responsible.

### A short region that is sufficient to activate gene expression in bundle sheath and veins

**Fig. 2.**
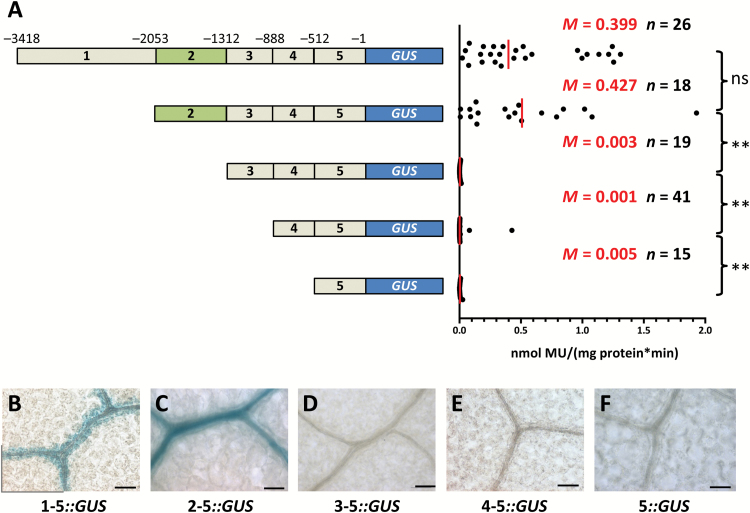
A 715-nucleotide region in the *SULTR2;2* promoter that is necessary for bundle sheath and veinal expression. (A) Schematic representation of the 5′ deletion series (left) with GUS activities (right). (B–F) GUS accumulation from each deletion. Deletion of region 2 abolished accumulation of GUS. Data from GUS activity assays include the median (*M*) indicated by red lines and the number (*n*) of independent lines. Statistical significance for each pairwise comparison is marked by a bracket to the right (ns, non-significant; **P<0.01). Histological GUS assays were allowed to proceed for 23 h (B), 4 h (C) and 6 days (D–F). Scale bars: 50 µm.

To better understand sequences that generate expression in BS and veinal cells, a 5′ deletion series was generated (Fig. 2A). The nucleotides downstream of the predicted translational start site of *SULTR2;2* were not required to direct expression to BS and vasculature (Fig. 2A; Supplementary Fig S1 at *JXB* online). Subsequent deletions to the promoter were made and are hereafter referred to as regions 1–5. Removal of region 1 had no significant effect on either activity or spatial accumulation of GUS (Fig. 2A, C) indicating that no essential *cis*-regulatory elements are located within this section. Deletion of region 2 resulted in total loss of GUS activity and staining (Fig. 2A, D), and removal of regions 3 and 4 had no further effect (Fig. 2A, E, F). These data therefore indicate that nucleotides in region 1 do not impact on promoter activity, but that region 2 is necessary for BS and veinal expression in mature leaves. A separate deletion series involving slightly smaller regions indicated that the construct containing nucleotides from −2815 to +123 led to stronger GUS activity than those from −3418 to −1 (Supplementary Figs S1, S2). This indicates that either there is a repressor within nucleotides −3418 to −2815, and/or that there is an enhancer within nucleotides −1 to +123. However, these quantitative regulators did not abolish expression in the BS and vasculature and so we did not dissect them further. Rather, subsequent analysis was focussed on region 2, which was necessary for expression in BS and veinal cells.

**Fig. 3.**
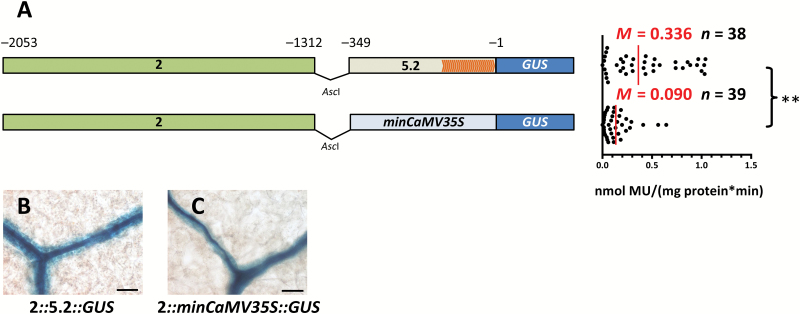
The *SULTR2;2* promoter contains a region that is sufficient to activate expression in bundle sheath and veinal cells. (A) Schematic representation of constructs containing nucleotides −2053 to −1312 combined with either the core promoter of *SULTR2;2* or the minimal CaMV35S promoter (left) and quantitative analysis of expression from each construct based on the GUS activity assay (right). Orange arrowheads within the core promoter indicate transcription start sites obtained by 5′-RACE. (B, C) Both constructs are sufficient to generate GUS accumulation in the bundle sheath. Data from GUS activity assays include the median (*M*) indicated by red lines and the number (*n*) of independent lines. Statistical significance is indicated to the right (**P<0.01). Histological GUS assays were allowed to proceed for 5 h (B) and 22 h (C). Scale bars: 50 µm.

The lack of GUS accumulation in the BS and veins, and loss of promoter activity once region 2 is removed could be because this region contains *cis*-elements that generate expression specifically in these cells or because it drives ubiquitous expression but regions 3 to 5 contain elements that restrict activity to the BS and veins. To investigate this possibility, 5′ rapid amplification of cDNA ends was first used to define the transcriptional start site of *SULTR2;2* (Supplementary Fig. S3). No single strong transcription start site was detected but rather multiple transcripts were initiated from position -125 onwards (Supplementary Fig. S2). Nucleotides spanning −349 to −1 were therefore considered likely to be sufficient for transcriptional initiation and are hereafter referred to as the core promoter. Fusion of region 2 to this core promoter led to MUG conversion that was comparable to that from the full-length promoter (Figs 2A, 3A) and was also sufficient to direct accumulation of GUS to veins and the BS (Fig. 3B). To exclude the possibility that the core promoter includes *cis*-elements necessary for BS- and vein-specific expression, region 2 was also fused to the minimal CaMV35S promoter, which does not drive significant expression. Although this led to 4-fold lower GUS activity than the full-length promoter (Fig. 3A), GUS accumulation was still restricted to veins and the BS (Fig. 3C). We conclude that although nucleotides −349 to −1 are not able to generate expression in the BS and veins (Fig. 2F), they likely represent the core promoter of *SULTR2;2*. In contrast, nucleotides −2053 to −1312 are sufficient to restrict expression to the BS and vascular tissue of Arabidopsis.

### *AtSULTR2;2* contains multiple redundant sequences mediating expression in the bundle sheath and vein

**Fig. 4.**
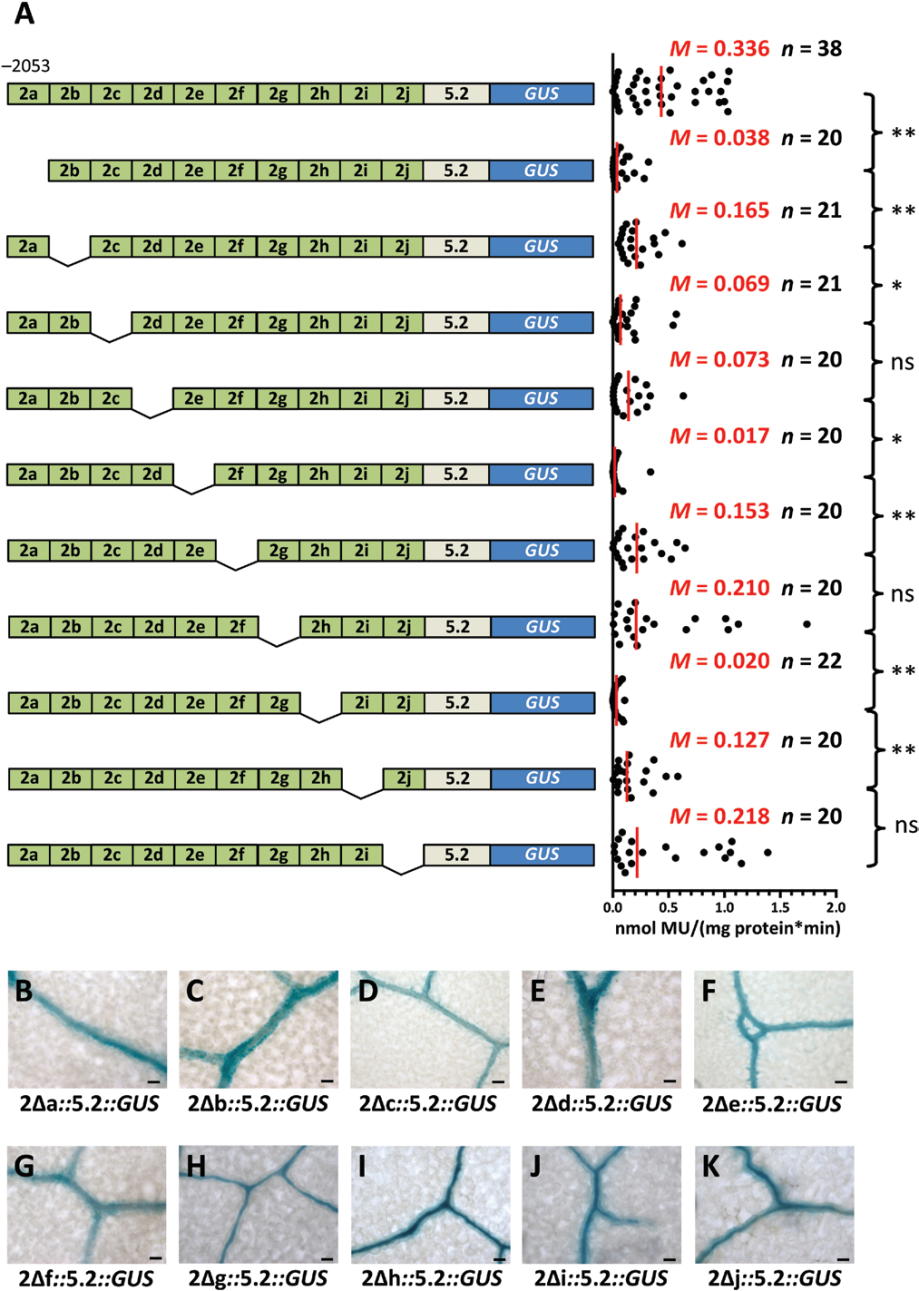
*SULTR2;2* contains a region with multiple redundant regions capable of directing bundle sheath and veinal expression. (A) Schematic representation of internal deletion series constructs (left) and quantitative analysis of expression from each construct based on the GUS activity assay (right). Except for the last deletion representing 66 bp, each construct lacks consecutive 75-bp sequences. These internal deletions modify the GUS activity (A), but none abolishes accumulation of GUS in the bundle sheath (B–K). Data from GUS activity assays include the median (*M*) indicated by red lines and the number (*n*) of independent lines. Statistical significance for each pairwise comparison is marked by a bracket to the right (ns, non-significant; *P<0.05; **P<0.01). Histological GUS assays were allowed to proceed for 23 h (B), 16 h (C), 48 h (D), 23 h (E), 48 h (F), 8 h (G), 3 h (H), 7 h (I), 3 h (J), and 19 h (K). Scale bars: 50 µm.

Having established that nucleotides −2053 to −1312 relative to the predicted translational start site are sufficient for BS and vein expression, an unbiased approach to further dissect this region was adopted. Ten consecutive deletions were made and each was fused to the core promoter of *SULTR2;2* (Fig. 4A). Strikingly, none of these deletions resulted in total loss of GUS activity (Fig. 4A), nor was GUS staining lost from BS and veins (Fig. 4B–K), indicating that despite an absence of repeated *cis*-elements in this region, significant functional redundancy in regulatory elements must mediate this patterning of gene expression. However, it was notable that GUS activity declined to varying degrees compared with the intact region (Fig. 4A), suggesting that either these redundant *cis*-elements act additively, or that this region contains quantitative elements regulating gene expression.

**Fig. 5.**
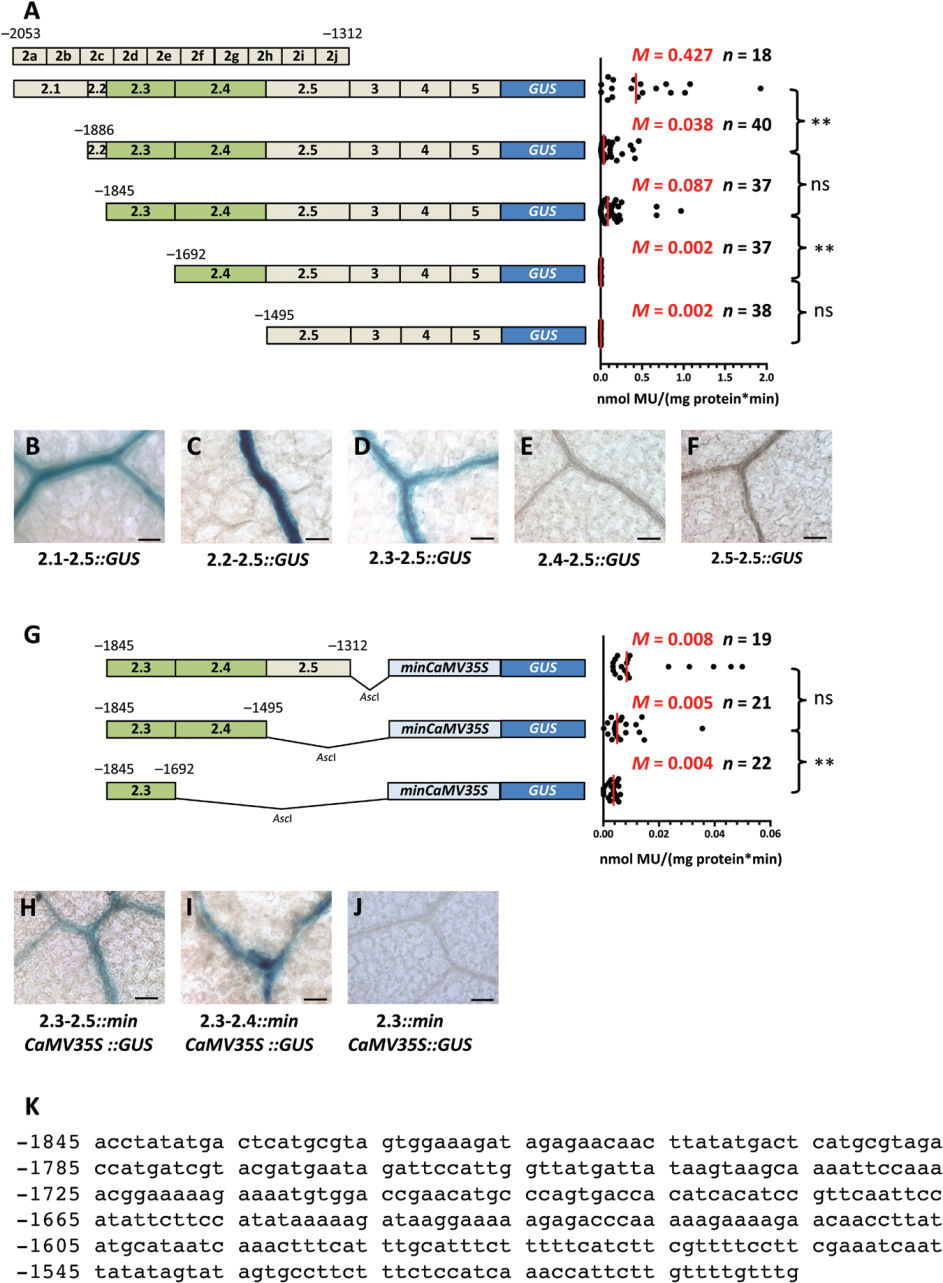
A short region of the *SULTR2;2* promoter that is necessary for expression in the bundle sheath and vein tissue. (A) Schematic representation of deletions made to region 2 of the *SULTR2;2* promoter (left) and quantitative analysis of expression from each construct based on the GUS activity assay (right). Activity was no longer detectable when subregions 4 and 5 were removed. (B–F) Histochemical staining of leaves indicated that subregion 3 is required for expression in the bundle sheath. (G) Schematic representation of 3′ deletion constructs placed upstream of the minimal CaMV35S promoter (left) and quantitative analysis of expression from each construct based on the GUS activity assay (right). (H–I) Nucleotides from −1845 to −1495 are sufficient to drive expression in the bundle sheath of leaves (H) and cotyledons (I), respectively. (J) Deletion of nucleotides −1692 to −1312 abolishes accumulation of GUS in the BS. Data from GUS activity assays include the median (*M*) indicated by red lines and the number (*n*) of independent lines. Statistical significance for each pairwise comparison is marked by a bracket to the right (ns, non-significant; **P<0.01). (K) Sequence of the 350 bp region that is sufficient for expression in bundle sheath strands of Arabidopsis. Histological GUS assays were allowed to proceed for 4 h (B), 47 h (C), 4 h (D) 6 d (E, F), 5 d (H), 2 d (I), and 29 h (J). Scale bars: 50 µm.

To better understand the extent to which *cis*-elements in this section of the promoter act redundantly, larger deletions were made from position −2053 (Fig. 5A). This generated five regions, hereafter referred to as subregions 2.1–2.5. Deletion of subregion 2.1 resulted in a strong reduction of GUS activity (Fig. 5A) implying that this region contains a quantitative enhancer element. However, accumulation of GUS was maintained in the BS and veins (Fig. 5C). Deleting subregion 2.2 had no clear additional impact on either this spatial patterning or activity (Fig. 5A, D). However, the subsequent deletion of subregion 2.3 caused loss of GUS activity and also loss of GUS staining in BS and vascular tissue (Fig. 5A, E). These data indicate that *cis*-regulatory elements mediating BS and vein expression are situated in subregion 2.3, or that quantitative elements in this region mask qualitative elements in distal subregions. To address these options, 3′ deletions of region 2 were also created (Fig. 5G). As subregions 2.1 and 2.2 had little impact on expression in the BS and vasculature, the last three subregions were fused to the minimal CaMV35S promoter. Expression from each of these three constructs was low (Fig. 5G); however, expression could be observed from the construct containing subregions 2.3, 2.4, and 2.5 (Fig. 5H). Removal of subregion 2.5 resulted in loss of GUS in rosette leaves although cotyledons still showed patchy staining restricted to the BS and vascular tissue (Fig. 5I). Once subregion 2.4 was removed, GUS activity was further reduced and GUS staining was no longer detectable even in seedlings (Fig. 5G, J). We conclude that subregion 2.3 contains *cis*-regulatory elements necessary (Fig. 5E) and regions 2.3 and 2.4 (Fig. 5I) are sufficient for expression of *AtSULTR2;2* in the BS and veins.

**Fig. 6.**
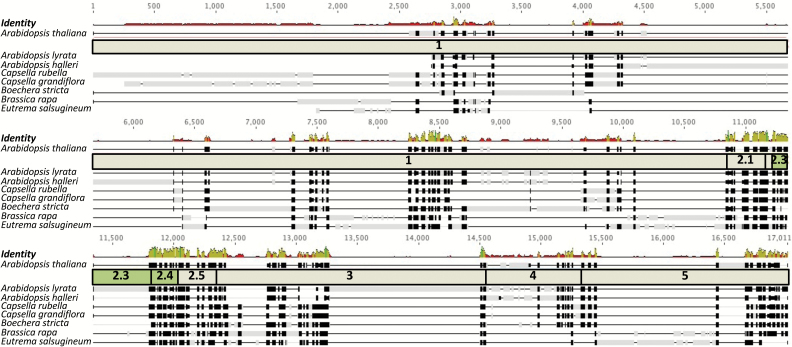
The region necessary for bundle sheath and veinal expression of *SULTR2;2* is highly conserved in the Brassicaceae. The promoter of *AtSULTRT2;2* was aligned against sequences ~5 kb upstream of genes from seven additional species of the *Brassicaceae*. With the exception of *Arabidopsis lyrata*, which contains a 446 bp insertion, region 2 is highly conserved. Black boxes indicate strong similarity of sequences, grey boxes sequences not matching the consensus sequence, and black lines gaps. The level of similarity is also indicated above the alignment. High peaks in green mark strong similarity, low peaks in red, poor similarity.

The *cis*-regulatory elements necessary for expression of *AtSULTR2;2* in BS and veins therefore appear to be located in a 350-nucleotide region of the promoter. As finer-scale deletions had failed to identify the exact *cis*-elements responsible for this phenotype (Fig. 4) a phylogenetic approach was undertaken. Orthologues of *AtSULTR2;2* were identified from seven species of the Brassicaceae. Alignments of sequences 5 kb upstream of each orthologue indicated that with the exception of *Arabidopsis lyrata*, which contains a 446 nucleotide insertion, region 2 is highly conserved (Fig. 6). However, no short sequences or motifs within this sequence that may restrict expression to the BS specifically could be identified. Although the results of this alignment therefore do not identify a specific *cis*-element that could be bound by a transcription factor responsible for generating expression in the BS and vein, they do support the functional analysis and implicate subregions 2.3 and 2.4 as critical components of the *SULTR2;2* promoter for this expression.

### The *AtSULTR2;2* promoter is capable of driving bundle sheath and vein expression in C_4_*Flaveria bidentis*

**Fig. 7.**
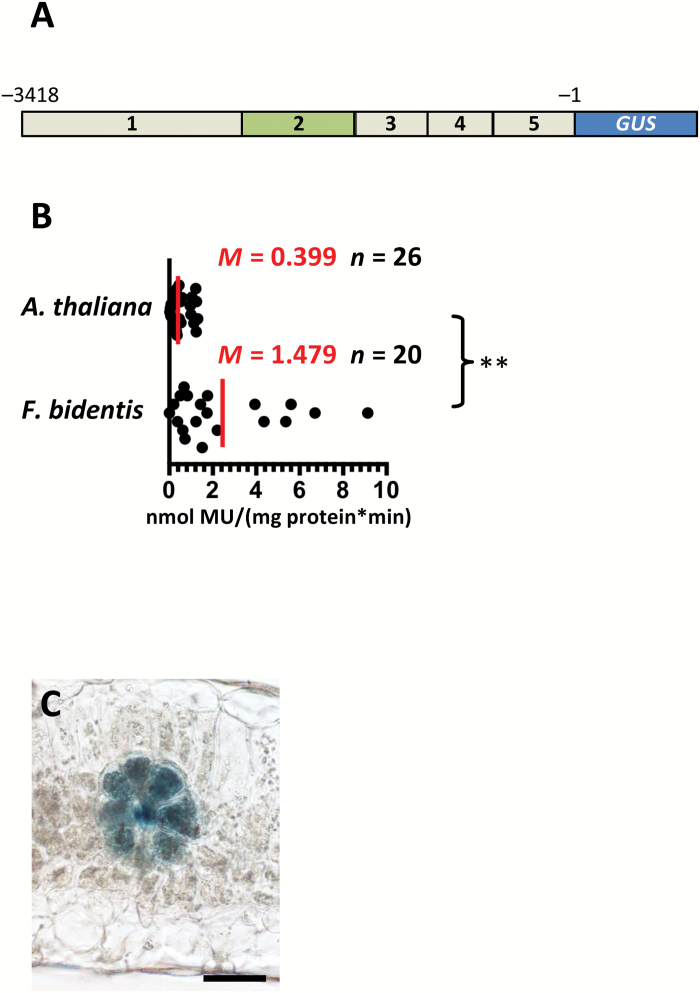
The Arabidopsis *SULTR2;2* promoter generates strong bundle sheath and vein expression in the C_3_ species *Flaveria bidentis*. (A) Schematic representation of the sequence placed into *Flaveria bidentis*. (B) Quantitative analysis of expression from each construct based on the GUS activity assay. To facilitate comparison, GUS data from the same construct in C_3_ Arabidopsis are included. (C) Representative image of transverse section of *Flaveria bidentis* after histochemical staining for GUS. Data from GUS assays include the median (*M*) indicated by red lines and the number (*n*) of independent lines. Statistical significance is indicated to the right (**P<0.01). Histological GUS assays were allowed to proceed for 4 h. Scale bar: 50 µm.

As the *GLDPA* promoter from the C_4_ species *F. trinervia* is able to confer BS and veinal expression in C_3_ Arabidopsis (Engelmann *et al.*, 2008), we next tested whether the Arabidopsis *SULTR2;2* promoter would lead to expression in the BS and veins of C_4_*F. bidentis*. GUS activity in transgenic *F. bidentis* plants was about fourfold higher than that in Arabidopsis (Fig. 7A, B). However, histochemical analysis of mature leaves revealed a very similar expression pattern to that in Arabidopsis with strong GUS accumulation in BS and vascular tissue but not in mesophyll cells (Fig. 7C). This indicates that transcription factors from *F. bidentis* recognize *cis*-regulatory elements from the Brassicaceae that generate expression in both the BS and veinal cells. The most parsimonious explanation for this finding is that these sequences represent part of an ancient and conserved mechanism that restricts gene expression to BS and vasculature of dicotyledenous leaves.

## Discussion

### *SULTR2;2* contains a short region that activates expression in the bundle sheath and veins

*SULTR2;2* encodes a low affinity transporter that facilitates movement of sulphate from the vascular bundle to palisade cells in the leaf (Takahashi *et al.*, 2000). Consistent with this function, analysis of both GUS and the histone2B::YFP fusion indicated that the *SULTR2;2* promoter directs expression to veins as well as to BS cells. It was notable that none of the various deletions we made to this promoter led to expression being restricted to either veins or BS cells. The *GLDPA* promoter from *F. trinervia* also drives expression in both veins and BS cells of Arabidopsis (Engelmann *et al.*, 2008). These findings therefore imply that the *SULTR2;2* and *GLDP* genes may be controlled by gene regulatory networks that are shared by these cell types. It is possible that these cell types share some gene regulatory networks because they are derived from the same lineage (Dengler and Nelson, 1999; Soros and Dengler, 2001).

Within the *SULTR2;2* promoter, one specific region that impacted on gene expression in the BS and veins was identified. *SULTR2;2* is located directly upstream of a second sulphur transporter gene, *SULTR1;2*, which is also preferentially expressed in bundle sheath strands (Aubry *et al.*, 2014). Because they are transcribed from opposite strands it is possible that the regulatory mechanisms described here control the expression of both genes. The sequence identified, consisting of 350 nucleotides, is both necessary and sufficient for generating expression in BS and veins. No specific *cis*-elements that are repeated across this entire region were identified. Moreover, it was also not possible to identify sequences that were shared between this region and the small number of promoters (Wysocka-Diller *et al.*, 2000; Knappe *et al.*, 2003; Dhondt *et al.*, 2010; Wiludda *et al.*, 2012) that have been reported to drive expression in BS cells of Arabidopsis (Supplementary Fig. S4). Small consecutive deletions within this region failed to abolish this spatial patterning implying that multiple contiguous elements act redundantly to generate strong and stable expression in the BS and vasculature. The only detectable impact of deleting any part of this region was for strength of expression to be reduced. We therefore propose that either multiple independent modules contained within this region act additively, or that distinct quantitative elements are co-located, and at least partially overlapping, with *cis*-elements that determine this expression in BS and veinal cells. Redundancy of this sort has previously been reported for the promoter of *Phenylalanine Ammonia Lyase*2, which drives xylem-specific expression in tobacco (Leyva *et al.*, 1992; Hatton *et al.*, 1995), *DORNRÖSCHEN-LIKE* of Arabidopsis, which contains three functionally redundant enhancers (Comelli *et al.*, 2016), and *EVEN-SKIPPED (EVE*) from *Drosophila melanogaster*, where a minimal enhancer is sufficient to direct expression of *EVE* to the second stripe, but surrounding binding sites increase the robustness of this patterning during genetic and environmental perturbations (Ludwig *et al.*, 2011). Thus, although the exact role of redundancy in the regulation of *SULTR2;2* is unclear, it may also increase robustness in the control of gene expression during environmental perturbations, and/or increase patterning precision (Barolo, 2012; Payne and Wagner, 2015).

Compared with other examples of elements that restrict gene expression to BS and veinal cells of C_3_ species, this single block of sequence from *SULTR2;2* that acts as a positive regulator of transcription appears to operate via relatively simple mechanisms. For example, the *F. trinervia GLDPA* generates BS and vein expression (Engelmann *et al.*, 2008) because of a complex interplay between transcriptional and post-transcriptional processes. These are mediated by distal and proximal sequences relative to the translational start site, leading to repression of *GLDP* expression in mesophyll cells (Wiludda *et al.*, 2012). We therefore propose that the positive regulator located upstream of *SULTR2;2* could be used as a synthetic module to manipulate or engineer processes in BS and vein cells of Arabidopsis.

### The bundle sheath and vein element of *SULTR2;2* is conserved in the Brassicaceae and functional in the Asteraceae

Alignment of *SULTR2;2* promoters from multiple species of the Brassicaceae did not reveal an individual shared *cis*-element but rather highlighted a sequence that was conserved across the whole of region 2. This sequence conservation argues for relatively strong purifying selection compared with the rest of the *SULTR2;2* promoter and also implies that this region may function as part of a widely conserved positive regulator of gene expression in BS and veins of the Brassicaceae. Consistent with this proposal and indicating that these regulatory elements may be even more ancient, when the *SULTR2;2* promoter was placed into the phylogenetically distant C_4_ species *F. bidentis* it was also recognized by *trans*-factors that restricted gene expression to the C_4_ BS and vasculature. This is analogous to the behaviour of the *GLDPA* promoter from C_4_*F. trinervia*, which is able to restrict expression to the BS and veins of Arabidopsis (Engelmann *et al.*, 2008; Wiludda *et al.*, 2012). Currently, the crown ages of the rosids and asterids are estimated to be 108–117 and 107–117 million years ago (Wikström *et al.*, 2001; Sanderson *et al.*, 2004) indicating that these clades diverged in the early Cretaceous. Whilst for both *SULTR2;2* and *GLDP* it is possible that different mechanisms lead to BS and vein expression in species of the Brassicaceae and Asteraceae, it seems more likely that expression of each gene is determined by ancient and highly conserved *cis*-regulatory codes that have been maintained since these clades diverged from their last common ancestor. Although the plant vasculature is thought to have started to evolve from 450 to 430 million years ago (Ye, 2002; Furuta *et al.*, 2014), to our knowledge there are no clear estimates of when the BS originated. It would be intriguing if regulatory networks operating in both the veins and BS cells are uncovered that can be associated with the evolution of the vasculature in early diverging lineages of land plants.

## Supplementary data

Supplementary data are available at *JXB* online.

Fig. S1. Nucleotides −3418 to −1 relative to the predicted translational start site generate preferential accumulation of the GUS reporter in bundle sheath cells.

Fig. S2. Additional deletion series generated for *SULTRT2;2*.

Fig. S3. Alignment of 5′ ends of cDNAs obtained via 5′ rapid amplification of cDNA ends.

Fig. S4. Dot plots indicating lack of conservation between the *SULTR2;2* promoter and others reported to drive expression in the bundle sheath of Arabidopsis.

## Supplementary Material

Supplementary Figures S1-S4
