# Supplementary material for: Expression of SULTR2;2, encoding a low-affinity sulphur transporter, in the Arabidopsis bundle sheath and vein cells is mediated by a positive regulator
Source: J Exp Bot. 2018 Jul 19;69(20):4897–906. doi: 10.1093/jxb/ery263 (PMC6137973; doi:10.1093/jxb/ery263)
Supplement: Supplementary Figures S1-S4 [file ery263_suppl_supplementary_figures_s1-s4.pdf]

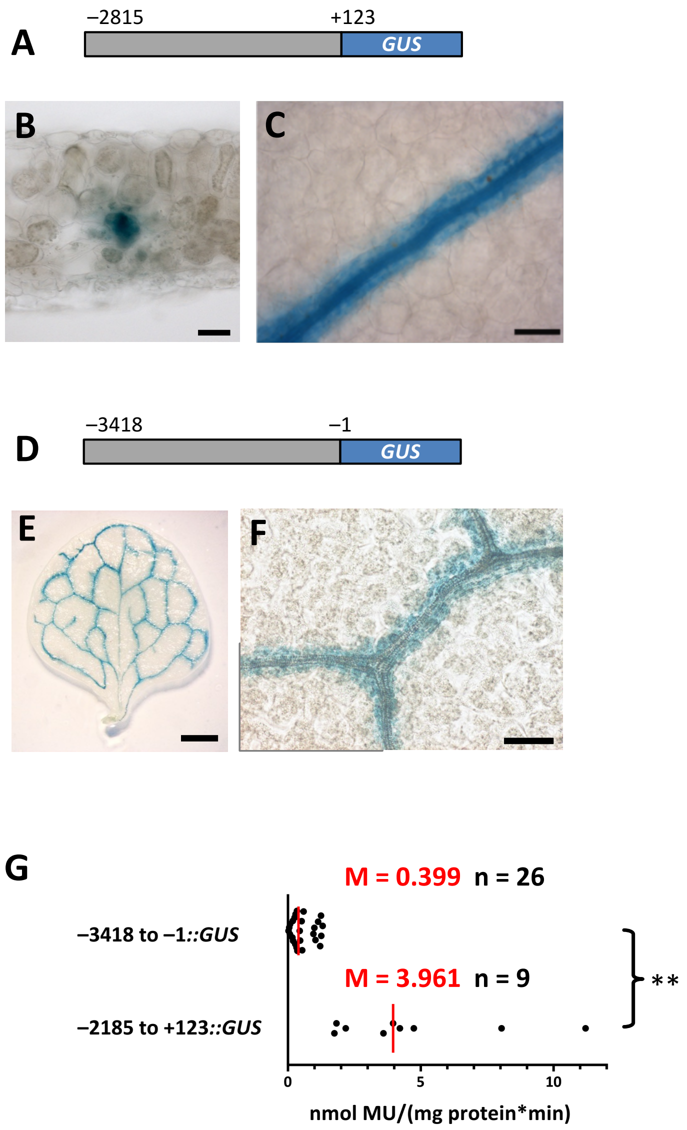


**Figure S1:** Nucleotides -3418 to -1 relative to the predicted translational start site generate preferential accumulation of the GUS reporter in bundle sheath cells. **(A&D)** Schematics of the constructs used. **(B,C,E,F)** Representative images of each reporter indicating preferential GUS staining in the bundle sheath and vasculature. **(G)** Quantitative analysis of expression from each construct based on the GUS activity assay. Statistical significance is marked by a parenthesis to the right, ** =p<0.01. Scale bars represent 50 µm (B,C,F) and 500 µm (E).

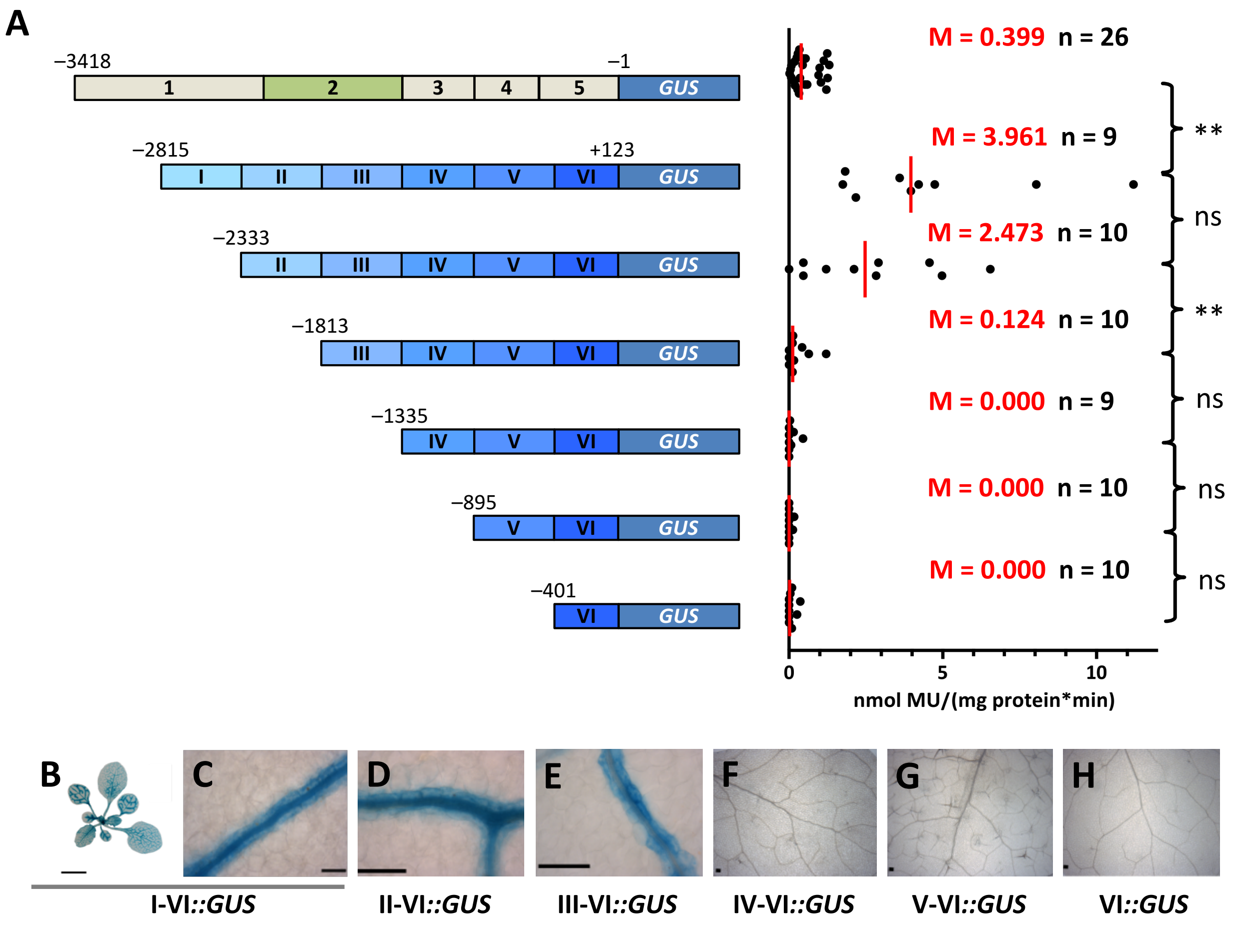


**Figure S2: Additional deletion series generated for *SULTRT2;2*. ( A)** Schematics illustrating each deletion (left) and quantitative analysis of expression from each construct based on the GUS activity assay (right). The series was designed to remove around 400 bp each time and to avoid *cis*4 regulatory elements predicted by the software PLACE. Deletion of region II resulted in a decline in GUS activity and deletion of region III lead to a total loss of expression. **(BD H)** Representative images after histological staining for GUS indicating that deleting region III led to loss of GUS in the bundle sheath. Data from GUS activity assays include the median (M) indicated by red lines and the number (n) of independent lines. Statistical significance for each pairwise comparison is marked by a parenthesis to the right (non-significant = n s, * * = p<0.01). H istological G US a ssays were allowed to proceed for 6 h. Scale bars each 50 µm.

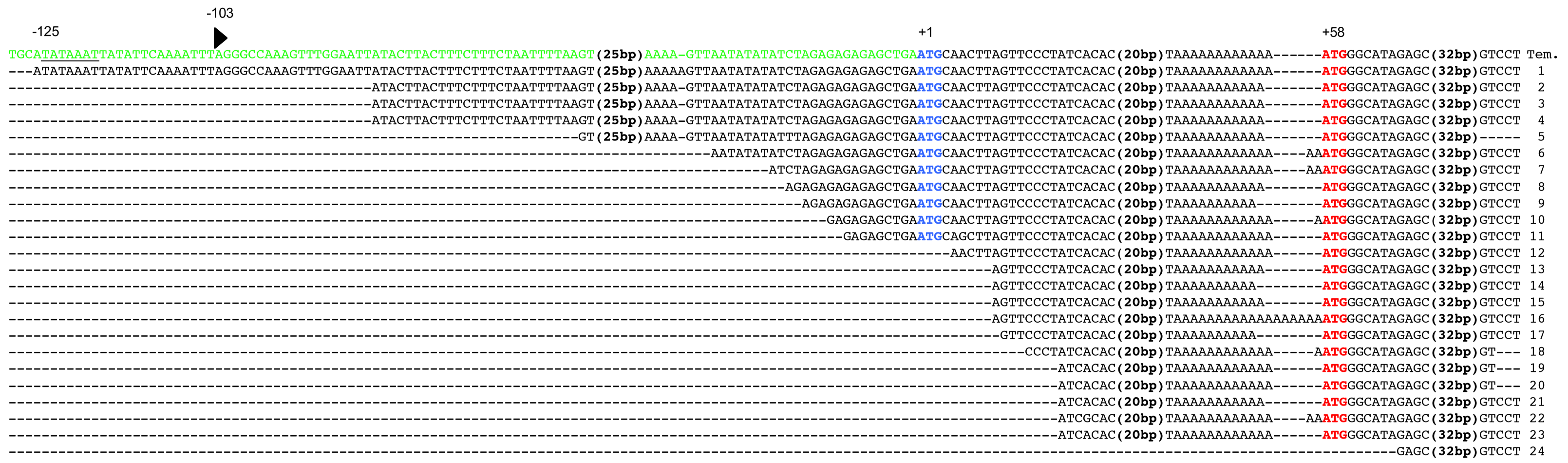

**Figure S3:** Alignment of 5’ ends of cDNAs obtained via 5’ Rapid amplification of cDNA ends. No distinct transcription start site was found. The top line shows the template used for alignment. Predicted translational start sites are marked in blue (annotated in TAIR Accession 1009028759) and red (from Takahashi *et al.* (2000)). A TATA box motif found by the PLACE database is underlined. The black arrowhead marks the TAIR annotated transcription start site (TAIR Accession 1009028759).

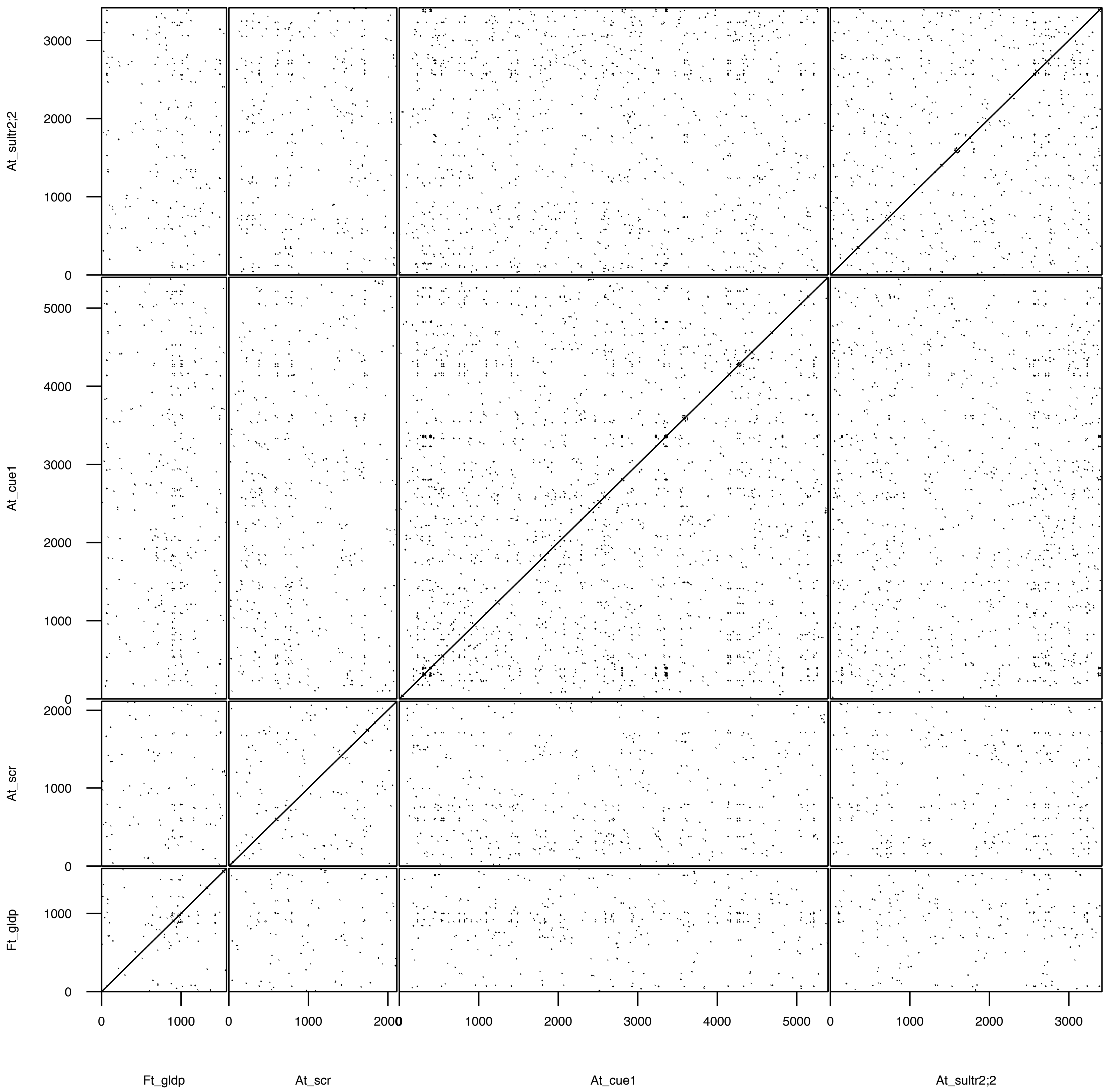


**Figure S4:** Dot plots indicating lack of conservation between the *SULTR2;2* promoter and others reported to drive expression in the bundle sheath of *A. thaliana*. The *SULTRT2;2* promoter was compared with the *Flaveria trinervia GLDP*, as well as the *A. thaliana SCR* and *CUE1* promoters. Each dotplot is based on a comparison with word size eight. The straight and continuous diagonal line running left to right indicates identical sequences when each promoter is compared with itself. The absence of additional diagonal lines indicates no co-linear conserved regions are shared between promoters.
